# Diagnostic value of serum LDH in children with refractory Mycoplasma pneumoniae pneumoniae: A systematic review and meta-analysis

**DOI:** 10.3389/fped.2023.1094118

**Published:** 2023-03-20

**Authors:** Shumin Wang, Zhiyan Jiang, Xuejun Li, Chenghui Sun, Yixing Zhang, Zhen Xiao

**Affiliations:** Pediatrics, Longhua Hospital Affiliated to Shanghai University of Traditional Chinese Medicine, Shanghai, China

**Keywords:** lactate dehydrogenase, pneumonia, mycoplasma, diagnostic testing, meta-analysis

## Abstract

**Background:**

To investigate the relationship between serum Lactate dehydrogenase (LDH) and refractory Mycoplasma pneumoniae pneumonia (RMPP) in juvenile individuals.

**Methods:**

Search Chinese databases and English databases. The retrieval time limit is from the establishment of the database to 2022-04-27. And screening and inclusion of relevant diagnostic test literature. The QUADAS-2 method was used to evaluate the quality of the included literature. The random effects model was used to combine sensitivity, specificity, likelihood ratio, diagnostic odds ratio, summary receiver operating characteristic curve, and area under summary receiver operating characteristic curve to evaluate the prediction value of LDH for RMPP. Subgroup analyses were used to explore sources of heterogeneity.

**Results:**

① A total of 29 literatures that met the criteria were included in the study, and the quality of the literature was medium and high, with a total of 702,2 patients. ② The combined sensitivity, specificity, positive likelihood ratio, negative likelihood ratio, diagnostic odds ratio, and area under the curve of the studies were: 0.75 (95% *CI* = 0.73–0.76), 0.73 (95% *CI* = 0.72–0.74), 3.61 (95% *CI* = 2.86–4.56), 0.30 (95% *CI* = 0.23–0.39), 13.04 (95% *CI* = 8.24–20.63), and 0.85(95% *CI* = 0.82–0.88). ③ The results of subgroup analysis showed that Compared with the subgroup with LDH threshold ≤400 IU/L, the AUC increased from 0.84 (95% *CI* = 0.80–0.87) to 0.89 (95% *CI* = 0.86–0.91).

**Conclusions:**

The serum LDH has good accuracy for the diagnosis of RMPP and can serve as a diagnostic marker for RMPP.

## Background

Mycoplasma pneumoniae (MP) is a small inactive growing microorganism between bacteria and virus, spread mainly by droplets, which is an important cause of community-acquired pneumonia (CAP) (1). MP can cause up to 20%–40% of CAP in the general population during epidemics, rising to as much as 70% in closed populations (2). Despite the fact that MP is self-limiting, some clinical cases develop refractory Mycoplasma pneumoniae pneumonia (RMPP), a serious and life-threatening infection. In addition to causing lung diseases such as lung abscess, bronchiolitis obliterans (BO), pulmonary embolism (PE) and pleural effusion, it can also induce extrapulmonary diseases such as encephalitis, Guillain-Barre syndrome (GBS), Henoch-Schönlein purpura (HSP), and Kawasaki disease (KD) (3–5). seriously affect the quality of life of children. In the past 10 years, especially in Asian countries, more and more RMPP have been reported. RMPP is defined as a case with prolonged fever accompanied by deterioration of radiological findings despite appropriate management with macrolide treatment for ≥7 days, which is usually diagnosed after onset, and therefore has a certain clinical hysteresis, the timing of treatment may be delayed (6–8). At the same time, the abuse of more advanced antibiotics and the irregular use of glucocorticoids often occur, which makes it urgent to find an early predictor of RMPP.

Lactate dehydrogenase (LDH), an inflammatory marker, is a major component after glycolysis. LDH exists in various organs of the human body, including heart, liver, lung, kidney, skeletal muscle, etc (9). When organs suffer from inflammation and other injuries, especially after lung tissue is damaged by hypoxia, LDH will spill over into the blood and other external spaces due to cell division or damage to cell membranes, resulting in an increase in the level of LDH in the blood (10). Mature children are more likely to be injured and cause LDH to rise. There has been an increase in clinical studies evaluating the diagnostic value of LDH for RMPP in recent years, but the quality of the literature is mixed, and no relevant meta-analysis has been published. Therefore, this article aims to collect relevant high-level literature evidence and combine the high-quality research results that have been published so far, in order to further study and clarify the practical application value and accuracy of LDH in the diagnosis of RMPP.

## Methods

This study was conducted in accordance with preferred reporting items for a systematic review and meta-analysis of diagnostic test accuracy studies (PRISMA-DTA) and we registered the review on PROSPERO (registration number: CRD42022336133) (11).

### Search strategy

We performed a search of the databases China National Knowledge Infrastructure (CNKI), Wanfang Database, Cqvip Database (VIP), SinoMed, PubMed, EMBASE, Cochrane Library, Web of Science databases without language restrictions from their inception to April 27, 2022. Taking PubMed as an example, the Medical Subject Headings (MeSH) terms and keywords used in the search were as follows: ((("Pneumonia, Mycoplasma"[Mesh]) OR ((((((((((((((((Refractory Mycoplasma pneumoniae pneumonia[Title/Abstract]) OR (Refractory Pneumonia, Primary Atypical[Title/Abstract])) OR (Refractory Atypical Pneumonia, Primary[Title/Abstract])) OR (Refractory Atypical Pneumonias, Primary[Title/Abstract])) OR (Refractory Pneumonias, Primary Atypical[Title/Abstract])) OR (Refractory Primary Atypical Pneumonia[Title/Abstract])) OR (Refractory Primary Atypical Pneumonias[Title/Abstract])) OR (Refractory Mycoplasma Pneumonia[Title/Abstract])) OR (Refractory Mycoplasma Pneumonias[Title/Abstract])) OR (Refractory Pneumonias, Mycoplasma[Title/Abstract])) OR (Refractory Mycoplasma ovipneumoniae Infection[Title/Abstract])) OR (Refractory Mycoplasma ovipneumoniae Infections[Title/Abstract])) OR (Refractory Mycoplasma pneumoniae Infection[Title/Abstract])) OR (Refractory Mycoplasma pneumoniae Infections[Title/Abstract])) OR (Refractory Mycoplasma dispar Infection[Title/Abstract])) OR (Refractory Mycoplasma dispar Infections[Title/Abstract]))) AND (("L-Lactate Dehydrogenase"[Mesh]) OR (((((LDH[Title/Abstract]) OR (Dehydrogenase, L-Lactate[Title/Abstract])) OR (L Lactate Dehydrogenase[Title/Abstract])) OR (Lactate Dehydrogenase[Title/Abstract])) OR (Dehydrogenase, Lactate[Title/Abstract])))) AND (sensitiv*[Title/Abstract] OR sensitivity and specificity[MeSH Terms] OR (predictive[Title/Abstract] AND value*[Title/Abstract]) OR predictive value of tests[MeSH Terms] OR accuracy*[Title/Abstract]). The references of the included studies and existing systematic reviews were hand-searched to find additional relevant articles. The full search strategy is included in Supplementary Appendix S1.

### Eligibility criteria

① Patientor population: Children with Mycoplasma pneumoniae pneumonia; Intervention: Serum LDH testing; Comparison: RMPP compliant with the “gold standard”; Outcome: Final diagnosis of RMPP. ② Studies evaluating LDH as a predictor of RMPP in children. ③ The serum LDH was determined before the diagnosis was made and the corresponding threshold was determined to further evaluate the actual accuracy of LDH for predicting RMPP after confirming or excluding RMPP according to the “gold standard”. ④ The results of the literature must provide sufficient data to construct a 2 × 2 contingency table to accurately calculate the specific values of true positive (TP), false positive (FP), false negative (FN), and true negative (TN) cases when LDH predicted RMPP in this study. ⑤ The type of study design is prospective or retrospective study. ⑥ The study also needs to clearly point out the reference standard of RMPP.

### Exclusion criteria

① conference abstracts or study protocols. ② duplicate publications. ③ studies with incomplete data or no relevant outcome.

### Research selection

Two reviewers (Shumin WANG and Xuejun LI) independently screened the literature. Initially, duplicate and irrelevant publications were excluded based on their title and abstract. Later, each of them independently read the full text of each potentially eligible article and finally identified all studies. In case of disagreement, discussions were conducted with a third investigator (Yixing ZHANG) until consensus was reached.

### Data extraction

Two reviewers (Shumin WANG and Xuejun LI) independently extracted the followingdata using predesigned forms according to the guideline fordata extraction for systematic reviews and meta-analysis (12), including the following information: first author, year of publication, nation, study type, sample size, gender ratio, average age of participants, “gold standard” for diagnosing RMPP, TP, FP, FN, and TN cases, thresholds of LDH. If the data in the literature is incomplete or cannot be directly extracted, the corresponding author will be contacted by email for consultation, and the literature that has not received an affirmative reply from the author will be excluded.

### Risk-of-bias assessment

The Quality Assessment of Diagnostic Accuracy Studies-2 (QUADAS-2) was used to assess the quality of the included studies (13). The tool evaluates 4 domains: patient selection, index test, reference standard, and flow and timing. For each domain, the risk of bias is analyzed using different signaling questions. Beyond the risk of bias, the tool also evaluates the applicability of each included study to the research question for every domain.

### Statistical analysis

Meta-analysis was performed using The Stata software (version 14.0) and Meta-disc 1.4 software. Pooled sensitivity, specificity, positive likelihood ratio (PLR), negative likelihood ratio (NLR), and diagnostic odds ratio (DOR) were calculated using a bivariate mixed-effects model, and a comprehensive receiver operating characteristic curve (SROC) was drawn. Threshold effect was assessed by Spearman's correlation coefficient between the log value of sensitivity and the log value of specificity, when *P *< 0.05, it was considered to have a threshold effect. Heterogeneity was assessed using the *I*^2^ value, when *I*^2^ > 50%, indicating that the heterogeneity between studies was large. We performed subgroup and sensitivity analyses to verify the robustness of the overall results and explore the sources of heterogeneity. Subgroup analysis was performed according to factors such as different study sources, study type, mean age, gold standard, and threshold. Publication bias was assessed with Deeks' funnel plot.

## Results

### Search results

**Figure 1:**
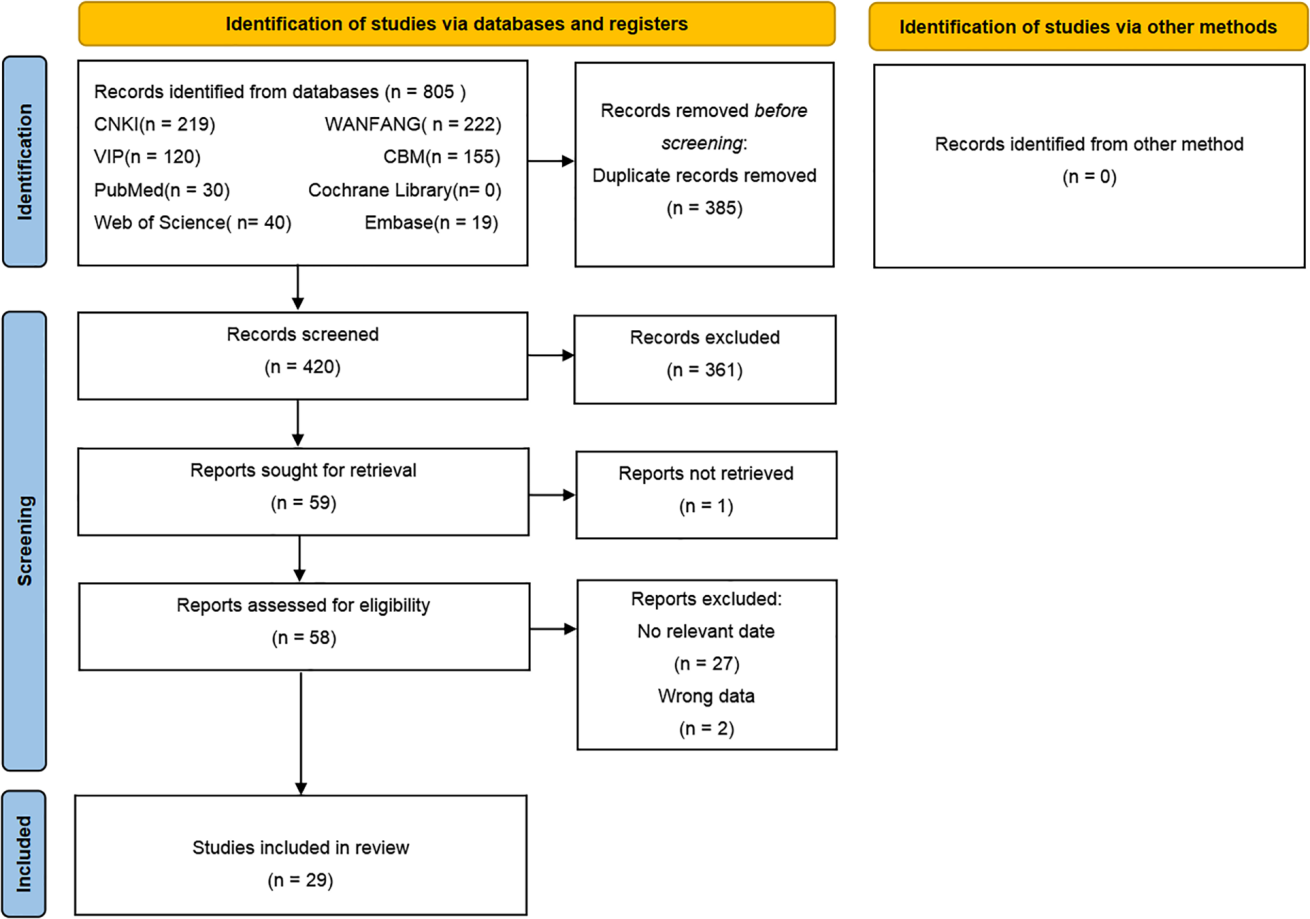
PRISMA flow chart of literature screening.

We acquired a total of 805 articles from the initial search of which 385 duplicate articles were excluded. Furthermore, 361 unrelated articles were excluded after the title and abstract screening and then 59 articles after full-text reading, Among of them, 1 articles could not obtain the full text, 27 articles could not extract relevant data, 2 articles have incorrect data. Finally, 29 studies were included in this review. The search selection process is shown in Figure 1.

### Characteristics of studies

A total of 29 articles were included, including 702,2 subjects, of which 227,9 children were diagnosed RMPP and 474,3 children were diagnosed general Mycoplasma pneumoniae pneumonia (GMPP). 2 studies (14, 15) used diagnostic criterion 1 as the “gold standard” for diagnosing RMPP, and the rest of the studies used diagnostic criterion 2 as the “gold standard” for diagnosing RMPP. The included studies were mainly from Asian countries, of which 1 study (16) was from Japan, 3 studies (14, 15, 17) were from South Korea, and the rest were from China. The sample size of each group ranged from 20 to 703 cases. 2 cohort studies (7, 18) and the rest were case-control studies. The basic characteristics of the included literature are shown in Table 1.

**Table 1:** Basic characteristics of the included studies.

First author	Year	Nation	Study type	Sample size	Gender ratio (male %)	Average age (year)	Gold standard	TP	FP	FN	TN	Threshold (IU/L)	Reference
Young-Jin Choi	2019	Korea	Case-control	123	57	5.00	1	11	22	4	86	350	(15)
Lilin Huang	2021	China	Case-control	72	58	1.95	2	14	20	8	30	365.5	(19)
Min Sik Jang	2021	Korea	Case-control	215	51	5.55	1	51	13	28	123	NR	(14)
Nan Li	2019	China	Case-control	174	90	4.85	2	26	75	32	41	315	(20)
Ta-Yu Liu	2018	China	Case-control	70	47	6.87	2	12	15	4	39	408	(21)
Aizhen Lu	2015	China	Cohort study	653	60	4.95	2	144	50	156	303	379	(7)
Nan Mu	2021	China	Case-control	82	NR	NR	2	19	8	9	46	340.77	(22)
Jun Wen	2021	China	Case-control	306	50	6.27	2	66	37	22	181	375.5	(23)
Jiangjiang Xu	2018	China	Case-control	703	49	4.95	2	106	267	46	284	335	(24)
Jiayu Zhai	2017	China	Case-control	628	56	5.74	2	116	164	26	322	402	(25)
Yuanyuan Zhang	2016	China	Case-control	634	55	3.90	2	116	169	29	320	417	(26)
Jiuling Zhao	2020	China	Case-control	154	58	9.05	2	35	41	10	68	436.5	(27)
Xuexiang Zheng	2020	China	Case-control	219	51	6.45	2	57	28	16	118	384.5	(28)
Norikazu Inamura	2014	Japan	Case-control	20	55	7.10	2	4	0	1	15	410	(16)
Eun Lee	2021	Korea	Case-control	149	52	5.85	2	14	73	4	58	NR	(17)
Zhen Wang	2017	China	Case-control	234	50	6.10	2	40	45	9	140	302	(29)
Shuang He	2020	China	Case-control	140	53	7.00	2	44	15	8	73	375.5	(30)
Xinhuan Shao	2015	China	Case-control	226	45	NR	2	58	12	10	146	353	(31)
Qin Zhou	2022	China	Case-control	152	53	5.90	2	46	14	26	66	432.5	(32)
Jindou Hao	2019	China	Case-control	137	53	6.05	2	44	20	23	50	314	(33)
Jianfang Zhang	2019	China	Case-control	317	61	7.80	2	76	64	32	145	360	(34)
Haiying Gao	2016	China	Cohort study	80	56	6.30	2	16	2	5	57	268	(18)
Xiaomei Liu	2020	China	Case-control	185	50	5.96	2	28	43	4	110	342.5	(35)
Ning Li	2017	China	Case-control	253	53	6.01	2	82	13	10	148	400.5	(36)
Shufen Mei	2018	China	Case-control	189	43	4.10	2	44	29	10	106	474.5	(37)
Zheng Guan	2020	China	Case-control	144	56	4.53	2	46	14	2	82	306.05	(38)
Fang Chen	2019	China	Case-control	147	57	6.04	2	45	7	9	86	130.1	(39)
Caili Liu	2020	China	Case-control	462	81	6.20	2	255	21	27	159	690	(40)
Jing Guo	2020	China	Case-control	154	60	6.35	2	85	7	9	53	691	(41)

NR is not reported; All included studies were first satisfied with MP-IgM positive or/and MP-DNA positive. diagnostic criteria 1 refer to “After 72 h the patients were checked for reduction of fever, cough, and crackles on the lung, as well as improvement of radiological findings. If there was no improvement, the patient was considered to have refractory MP pneumonia.” diagnostic criteria 2 refer to “(1) Prolonged fever for 7 days or more with appropriate macrolide antibiotic treatment. (2) Clinical (fever or respiratory symptoms such as cough, wheezing, and difficult breathing) and radiographic (pulmonary consolidation or pleural effusion) progression despite appropriate macrolide antibiotic treatment for at least 7 days.”

### Risk of bias assessment

**Figure 2:**
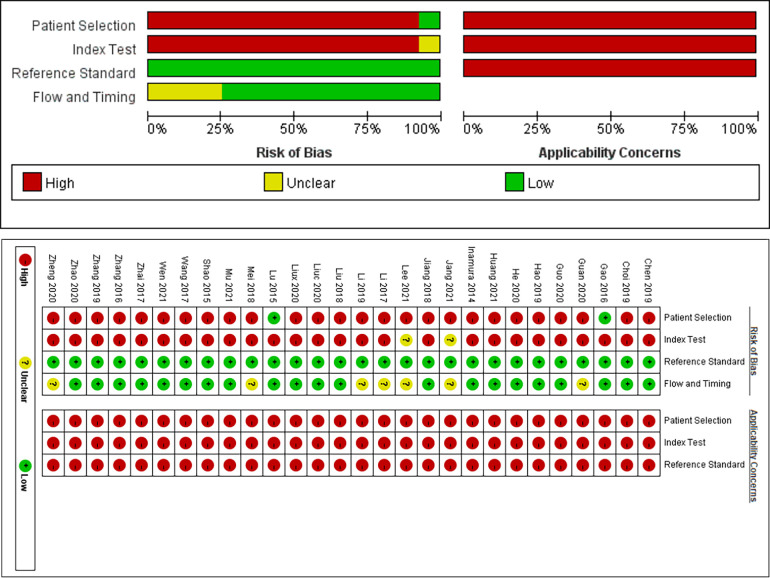
Quality assessment results of included studies based on QUADAS-2 tool criteria.

The quality of the included literature was evaluated based on the QUADAS-2 model, and the results are shown in Figure 2. 2 studies (7, 18) were clearly indicated as cohort studies with low risk of bias in patient selection, and the remaining studies were case-control studies, so there may be high risk of bias in patient selection. 2 studies (14, 17) were defined as uncertain risk in index test because they did not clearly state the threshold, and the remaining studies were defined as high risk of bias in index test because their threshold was not determined in advance. All studies were defined as low risk of bias with respect to the reference standard because each study clearly indicated the gold standard for diagnosis and the interpretation of the results was performed without knowledge of the results of the trial to be evaluated. 7 studies (14, 17, 20, 28, 36–38) did not specify the timing of LDH detection, so the flow and timing part was defined as uncertain risk, and the remaining studies were at low risk of bias. Overall, the quality of the included literature can be rated as moderate to high, and the results of all studies are generally credible.

### Analysis of diagnostic threshold

The data was imported into Meta DiSc1.4 software for analysis, and the Spearman correlation coefficient between the logarithm of sensitivity and (1-specificity) logarithm was −0.324 (*P *= 0.086 > 0.05), which was not significant, meaning that There was no threshold effect in this study. Further, by drawing a symmetrical SROC curve, there is no “shoulder-arm shape”, which further shows that this study has no threshold effect.

### Non-threshold effect heterogeneity

The Cochran-Q test of the DOR showed that Cochran-Q = 312.50, *P *< 0.001, which means that there is heterogeneity caused by non-threshold effect in this study. Further, the *I^2^* of sensitivity, specificity and PLR, NLR, and DOR were all greater than 50%. Therefore, the random effects model was used to combine the above five effect sizes.

### Evaluation metrics for diagnostic tests

**Figure 3:**
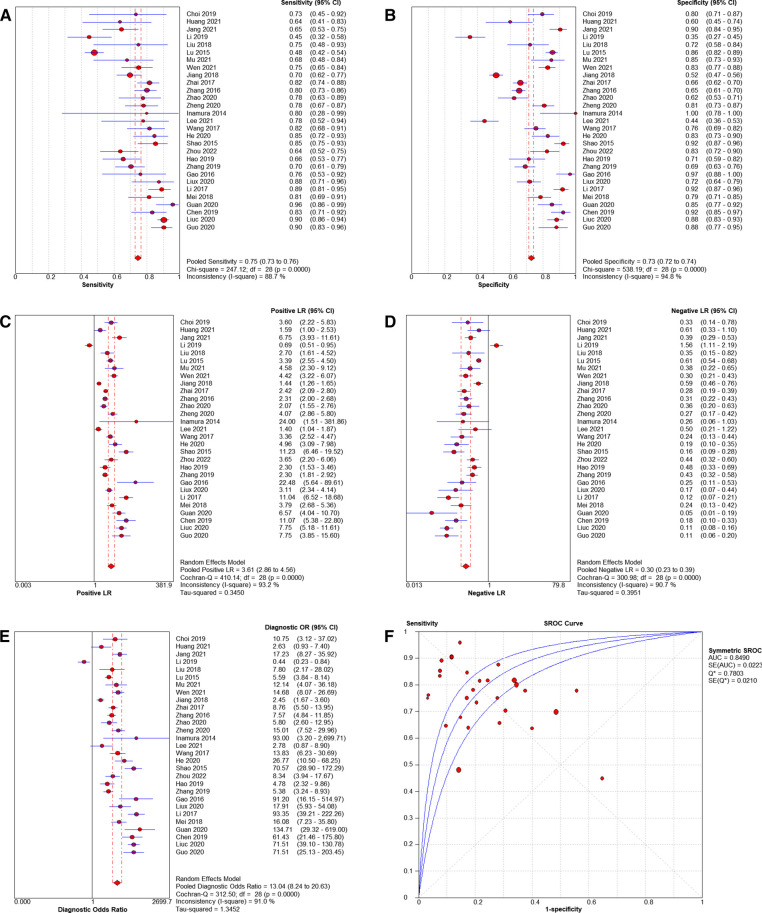
Forest plots of pooled sensitivity and specificity (**A, B**), Forest plots of pooled PLR and NLR (**C, D**), Forest plots of DOR (**E**) and SROC of diagnosis performance of included studies (**F**).

Pooled sensitivity was 0.75 (95% *CI* = 0.73–0.76), pooled specificity was 0.73 (95% *CI* = 0.72–0.74), pooled PLR was 3.61 (95% *CI* = 2.86–4.56), pooled NLR was 0.30 (95% *CI* = 0.23–0.39), pooled AUC = 0.85(95% *CI* = 0.82–0.88), Q index = 0.7803, and the combined DOR was 13.04 (95% *CI* = 8.24–20.63). Results shown in Figure 3.

### Sensitivity analysis

**Figure 4:**
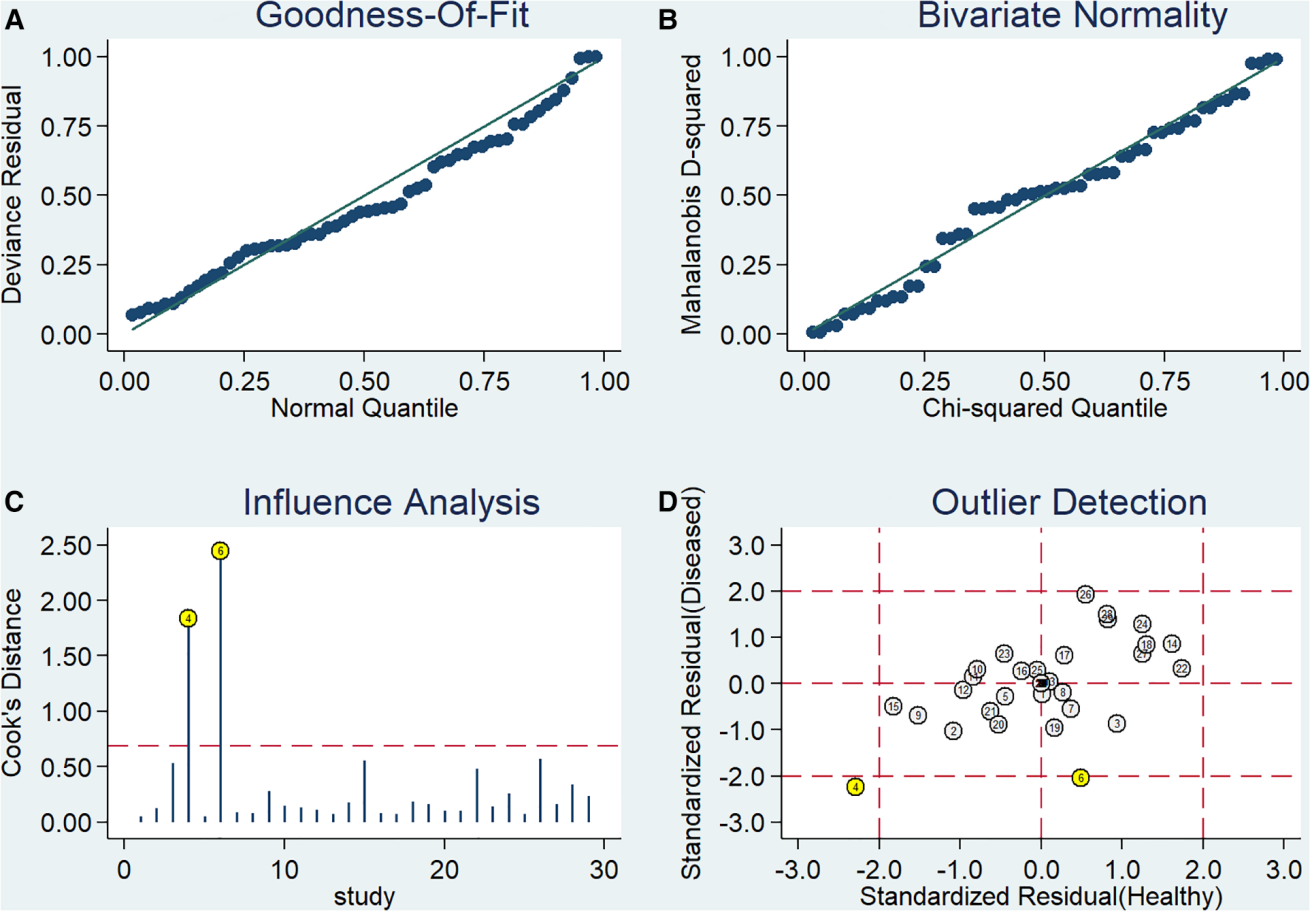
Sensitivity analysis.

Stata 14.0 was used to perform sensitivity analysis on the data of this study, and the results are shown in Figure 4. It can be clearly seen from the figure that there are 2 original studies (7, 20) with strong sensitivity, and other original studies will not cause the sensitivity of the calculation results. Therefore, the heterogeneity test was performed again after removing the above 2 studies. The Cochran-Q test of the DOR showed that Cochran-Q = 216.51, *P *< 0.001, suggesting that there is still heterogeneity. Pooled sensitivity was 0.80 (95% *CI* = 0.78–0.81), pooled specificity was 0.73 (95% *CI* = 0.71–0.74), pooled PLR was 3.86 (95% *CI* = 3.05–4.88), pooled NLR was 0.27 (95% *CI* = 0.22–0.34), pooled AUC = 0.86 (95% *CI* = 0.83–0.89), Q index = 0.7950, and the combined DOR was 15.36 (95% *CI* = 9.94–23.72). Similar to the results before exclusion. Therefore, the results of this study are relatively stable.

### Subgroup analysis

After the heterogeneity test of the results of the 29 studies, it was suggested that there was direct non-threshold heterogeneity among the studies. Therefore, we further performed subgroup analyses according to different study sources, study type, mean age, gold standard, and threshold. The results are shown in Table 2.

**Table 2:** Subgroup analysis results of the included studies.

Subgroup	Case	Sensitivity (95% *CI*)	Sensitivity *I*^2^ (95% *CI*)	Specificity (95% *CI*)	Specificity *I*^2^ (95% *CI*)	AUC (95% *CI*)
**Region**
Japan/Korea	4	0.69 (0.58–0.79)	0.00 (0.00–100.00)	0.83 (0.56–0.95)	97.45 (96.05–98.85)	0.75 (0.71–0.79)
China	25	0.78 (0.73–0.83)	91.14 (88.58–93.70)	0.79 (0.73–0.84)	95.93 (94.88–96.79)	0.85 (0.82–0.88)
China Northern city	8	0.78 (0.68–0.85)	87.30 (79.84–94.75)	0.77 (0.63–0.86)	95.61 (93.73–97.50)	0.84 (0.80–0.87)
China Southern city	17	0.78 (0.72–0.83)	92.61 (90.14–95.09)	0.80 (0.74–0.85)	96.23 (95.21–97.26)	0.86 (0.83–0.89)
**Average age (year)**
≤6	13	0.73 (0.64–0.81)	90.27 (86.20–94.34)	0.71 (0.61–0.80)	96.02 (94.75–97.29)	0.79 (0.75–0.82)
>6	14	0.82 (0.77–0.86)	73.66 (59.70–87.61)	0.84 (0.78–0.88)	88.14 (83.09–93.19)	0.89 (0.86–0.92)
**Study type**
Case-control	27	0.79 (0.74–0.83)	81.74 (75.49–87.99)	0.78 (0.72–0.83)	95.42 (94.38–96.46)	0.85 (0.82–0.88)
**Gold standard**
Diagnostic criteria 2	27	0.78 (0.73–0.83)	90.37 (87.63–93.12)	0.79 (0.73–0.84)	95.98 (95.10–96.86)	0.85 (0.82–0.88)
**Threshold (IU/L)**
≤400	17	0.75 (0.68–0.81)	89.66 (85.84–93.48)	0.79 (0.71–0.85)	96.49 (95.56–97.43)	0.84 (0.80–0.87)
>400	10	0.83 (0.78–0.88)	78.05 (64.83–91.28)	0.80 (0.72–0.87)	93.84(91.24–96.43)	0.89(0.86–0.91)

### Publication bias assessment

**Figure 5:**
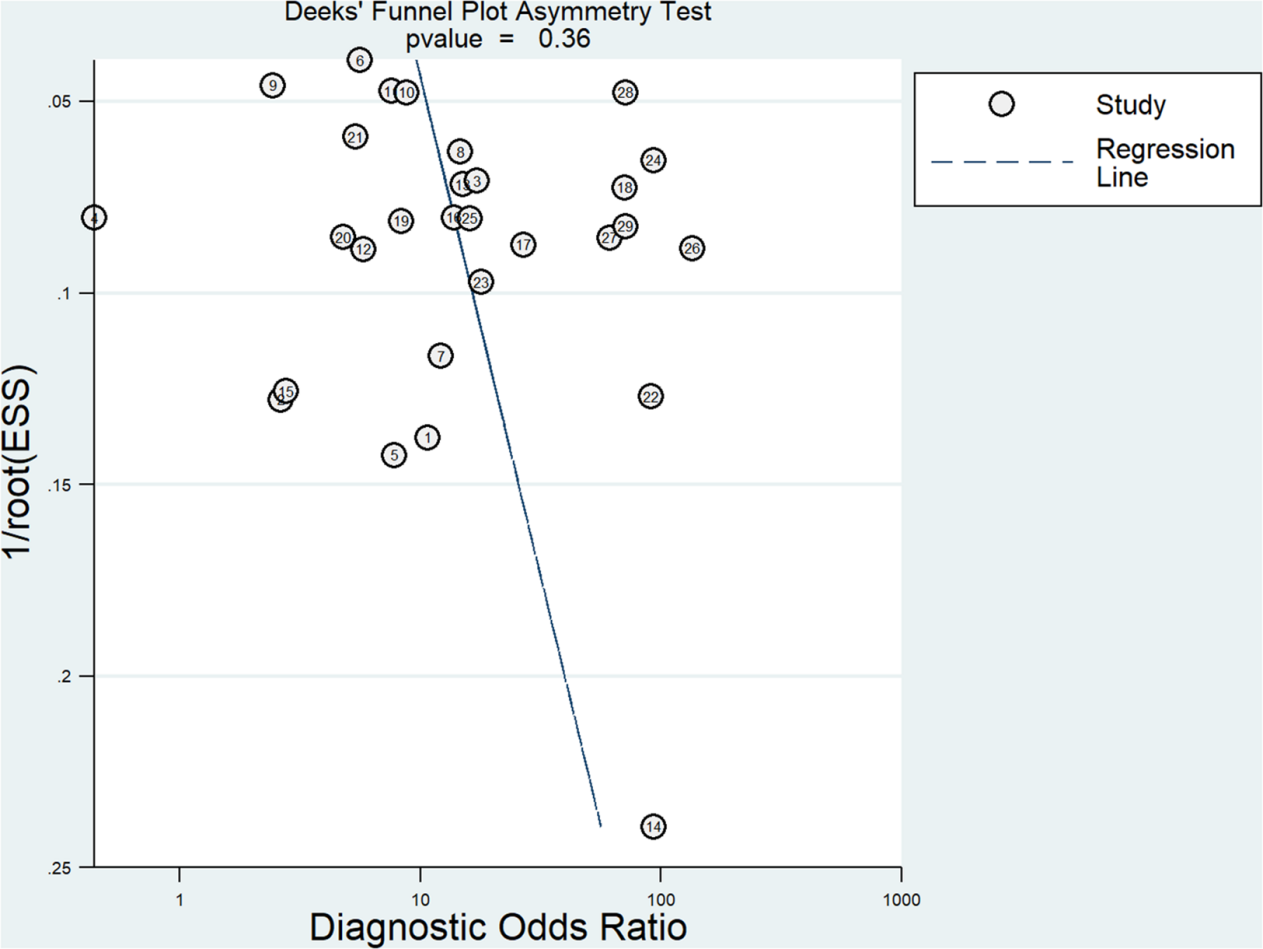
Deeks' funnel plot for evaluating publication bias among the included studies.

The Deeks' funnel plot showed that the included studies were approximately symmetrically distributed (*P *= 0.36), indicating that there was no significant publication bias. The results are shown in Figure 5.

## Discussion

Due to the particularity of MP structure and the limitations of children's own medication, macrolide antibiotics are still the first-line treatment drugs for the treatment of RMPP in children, such as azithromycin, erythromycin, etc. However, after infection with MP, the inflammatory response intensifies, secondary immune disorders occur, resulting in increased difficulty in the treatment of the disease, so glucocorticoids are added on the basis of antibiotic therapy to carry out anti-inflammatory, anti-allergic and immunosuppressive treatment to improve the quality of comprehensive intervention of the disease. RMPP is characterized by an overactive immune response to pathogens, in which case glucocorticoids are considered an immunomodulator that regulates the overactive host immune response. Although glucocorticoids have a good therapeutic effect on RMPP, for children, scientific dosage is equally important for the safety of treatment. A study indicated that children with RMPP who received methylprednisolone (1 mg/kg/dose, thrice daily for 3 days) recovered smoothly without any obvious side effects (42). Therefore, the appropriate prescription of antibiotics, as well as the rapid and accurate diagnosis of RMPP, is important. In a previous meta-analysis, inflammatory markers such as serum tumor necrosis factor-α (TNF-α) and interferon-*γ* (IFN-*γ*) could be used as diagnostic indicators of RMPP (43). However, due to its high cost and reliability that needs further clinical verification, it will be difficult to promote and popularize it in clinical practice in the next few years, especially in many primary hospitals. In contrast, the index of LDH is more widely used in clinical tests and carried out in medical institutions at all levels.

LDH was associated with many pulmonary diseases, such as obstructive diseases, microbial pulmonary diseases, and interstitial lung diseases (26). Tsai believes that high levels of LDH suggest refractory MPP and that systemic corticosteroids should be considered (42). This is consistent with the results of Inamura's study, who found that the sensitivity and specificity of LDH in the diagnosis of RMPP were 80% and 100%, respectively (16). Further studies indicated that among the LDH isoenzymes, the RMPP group showed significantly lower proportions of LDH1 and LDH2, and higher LDH4 and LDH5 percentage. Serum LDH4 plus LDH5 is a better biomarker than total LDH for the prediction of RMPP and use of systemic steroids (21). At the same time, serum LDH levels also predict the severity of RMPP. The AUC of LDH levels for RMPP children with severe chest radiography findings was 0.99, indicating a very high predictive validity for this index. A cut-off LD level of 530 IU/L showed very high sensitivity and specificity of 100% and 93%, respectively (44). And one study found that higher LDH levels on admission were significantly associated with a slow response to progressive treatment for MP pneumonia (17). However, some studies have pointed out that LDH has no advantage in predicting RMPP (20). Prior to our diagnostic meta-analysis, a risk factor-related meta-analysis by Huang discussed CRP, LDH, D dimer, etc. as risk factors for RMPP, but limited to one qualitative and only seven studies on LDH, with possible search bias (45). Therefore, we conducted a comprehensive search, we conducted a meta-analysis of the diagnostic efficacy of LDH on RMPP, and performed a subgroup analysis to classify and discuss different LDH thresholds, age, case sources, etc., hoping to better guide clinical practice.

This Meta-analysis included 29 studies involving a population of 702,2 cases. Firstly, we used QUADAS-2 to evaluate the quality of the included literature, which indicated that the quality of the literature was generally high. Therefore, the credibility of all research results was guaranteed to a certain extent. Secondly, we performed a non-threshold effect test using Meta DiSc 1.4 software, the Spearman correlation coefficient between the logarithm of sensitivity and (1-specificity) logarithm was −0.324 (*P *= 0.086 > 0.05), which was not significant, meaning that There was no threshold effect in this study. The non-threshold effect heterogeneity test was continued and found that the heterogeneity between studies was high (Cochran-Q = 312.50, *P *< 0.001). Pooled sensitivity was 0.75 (95% *CI* = 0.73–0.76), pooled specificity was 0.73 (95% *CI* = 0.72–0.74), pooled AUC = 0.85 (95% *CI* = 0.82–0.88) using random effects model pooled effect sizes. indicated LDH has good accuracy in diagnosing RMPP. Pooled PLR was 3.61 (95% *CI* = 2.86–4.56), pooled NLR was 0.30 (95% *CI* = 0.23–0.39), also suggesting that LDH can be used as a reliable indicator for predicting RMPP. In addition, we conducted a sensitivity analysis to find the source of heterogeneity, suggesting that there may be heterogeneity in 2 studies (7, 20), and performed a re-analysis after exclusion. Coincidentally, the results after exclusion were similar to those of previous studies (Before exclusion: pooled AUC = 0.85 (95% CI = 0.82–0.88) VS after exclusion: pooled AUC = 0.86 (95% CI = 0.83–0.89)), suggesting that the results of this study are relatively stable. At the same time, we conducted a subgroup analysis based on different study sources, study types, average age, gold standard and threshold. We found that LDH as a predictor of RMPP was more powerful in Chinese-derived studies compared with Japanese/Korea-derived studies. This situation may be due to differences in the diagnosis of RMPP among studies from different sources. In the subgroup with a mean age of more than 6 years, the sensitivity, specificity and AUC of LDH in the diagnosis of RMPP were 0.82 (95% *CI* = 0.77–0.86), 0.84 (95% *CI* = 0.78–0.88) and 0.89 (95% *CI* = 0.86–0.92), respectively. the diagnostic performance was higher than that of the subgroup with an average age of less than 6 years. The occurrence of these conditions may be related to the epidemiology of RMPP, which is most prevalent in school-age children (6–12 years). Compared with the subgroup with LDH threshold ≤400 IU/L, the AUC increased from 0.84 (95% *CI* = 0.80–0.87) to 0.89 (95% *CI* = 0.86–0.91). Finally, we performed the Deeks' funnel plot publication bias analysis of the included studies, suggesting that there was no publication bias.

Admittedly, our study also has the following limitations. First, although there are 29 studies included in this study, the included populations mainly involve the Asian region, where more RMPPs have been reported, and the applicability of the results to populations in other regions is unclear. Second, although a subgroup analysis was conducted based on differences in study sources, study type, mean age, gold standard, and threshold, there was still heterogeneity after combining the effect sizes, and the influence of the reasons for heterogeneity on the results should be considered when interpreting the results. Third, the LDH thresholds used among the included studies were not completely consistent. Although the Spearman correlation coefficient showed that there was no obvious threshold effect, different thresholds would still affect the accuracy of the combined results to a certain extent. Fourth, most of the included studies are case-control studies, which inevitably have population selection bias. In the future, more large-scale, multicenter prospective studies are needed to explore the accuracy of LDH in the diagnosis of RMPP.

In conclusion, the current evidence shows that serum LDH has good accuracy for the diagnosis of RMPP. Limited by the source and quality of the included population, more multi-center and high-quality studies need to be included in the future for verification.

## Data Availability

The raw data supporting the conclusions of this article will be made available by the authors, without undue reservation.
