# Supplementary material for: Diagnostic value of serum LDH in children with refractory Mycoplasma pneumoniae pneumoniae: A systematic review and meta-analysis
Source: Front Pediatr. 2023 Mar 20;11:1094118. doi: 10.3389/fped.2023.1094118 (PMC10067633; doi:10.3389/fped.2023.1094118)
Supplement: Supplementary file 1 [file Table1.docx]

*Supplementary Material*

Pubmed:

((("Pneumonia, Mycoplasma"[Mesh]) OR ((((((((((((((((Refractory Mycoplasma pneumoniae pneumonia[Title/Abstract]) OR (Refractory Pneumonia, Primary Atypical[Title/Abstract])) OR (Refractory Atypical Pneumonia, Primary[Title/Abstract])) OR (Refractory Atypical Pneumonias, Primary[Title/Abstract])) OR (Refractory Pneumonias, Primary Atypical[Title/Abstract])) OR (Refractory Primary Atypical Pneumonia[Title/Abstract])) OR (Refractory Primary Atypical Pneumonias[Title/Abstract])) OR (Refractory Mycoplasma Pneumonia[Title/Abstract])) OR (Refractory Mycoplasma Pneumonias[Title/Abstract])) OR (Refractory Pneumonias, Mycoplasma[Title/Abstract])) OR (Refractory Mycoplasma ovipneumoniae Infection[Title/Abstract])) OR (Refractory Mycoplasma ovipneumoniae Infections[Title/Abstract])) OR (Refractory Mycoplasma pneumoniae Infection[Title/Abstract])) OR (Refractory Mycoplasma pneumoniae Infections[Title/Abstract])) OR (Refractory Mycoplasma dispar Infection[Title/Abstract])) OR (Refractory Mycoplasma dispar Infections[Title/Abstract]))) AND (("L-Lactate Dehydrogenase"[Mesh]) OR (((((LDH[Title/Abstract]) OR (Dehydrogenase, L-Lactate[Title/Abstract])) OR (L Lactate Dehydrogenase[Title/Abstract])) OR (Lactate Dehydrogenase[Title/Abstract])) OR (Dehydrogenase, Lactate[Title/Abstract])))) AND (sensitiv*[Title/Abstract] OR sensitivity and specificity[MeSH Terms] OR (predictive[Title/Abstract] AND value*[Title/Abstract]) OR predictive value of tests[MeSH Terms] OR accuracy*[Title/Abstract])

EMBASE:

(('pneumonia,'/exp OR pneumonia,) AND ('mycoplasma'/exp OR mycoplasma) OR 'refractory mycoplasma pneumoniae pneumonia':ab,ti OR 'refractory pneumonia, primary atypical':ab,ti OR 'refractory atypical pneumonia, primary':ab,ti OR 'refractory atypical pneumonias, primary':ab,ti OR 'refractory pneumonias, primary atypical':ab,ti OR 'refractory primary atypical pneumonia':ab,ti OR 'refractory primary atypical pneumonias':ab,ti OR 'refractory mycoplasma pneumonia':ab,ti OR 'refractory mycoplasma pneumonias':ab,ti OR 'refractory pneumonias, mycoplasma':ab,ti OR 'refractory mycoplasma ovipneumoniae infection':ab,ti OR 'refractory mycoplasma ovipneumoniae infections':ab,ti OR 'refractory mycoplasma pneumoniae infection':ab,ti OR 'refractory mycoplasma pneumoniae infections':ab,ti OR 'refractory mycoplasma dispar infection':ab,ti OR 'refractory mycoplasma dispar infections':ab,ti) AND ('l lactate' AND dehydrogenase OR 'ldh':ab,ti OR 'dehydrogenase, l-lactate':ab,ti OR 'l lactate dehydrogenase':ab,ti OR 'lactate dehydrogenase':ab,ti OR 'dehydrogenase, lactate':ab,ti) AND ('sensitiv':ab,ti OR 'sensitivity and specificity':ab,ti OR 'predictive':ab,ti OR 'predictive value of tests':ab,ti OR 'accuracy':ab,ti)

Cochrane:

#1:Pneumonia, Mycoplasma

#2:(Refractory Mycoplasma pneumoniae pneumonia):ab,ti,kw OR (Refractory Pneumonia, Primary Atypical):ab,ti,kw OR (Refractory Atypical Pneumonia, Primary):ab,ti,kw OR (Refractory Atypical Pneumonias, Primary):ab,ti,kw OR (Refractory Pneumonias, Primary Atypical):ab,ti,kw OR (Refractory Primary Atypical Pneumonia):ab,ti,kw OR (Refractory Primary Atypical Pneumonias):ab,ti,kw OR (Refractory Mycoplasma Pneumonia):ab,ti,kw OR (Refractory Mycoplasma Pneumonias):ab,ti,kw OR (Refractory Pneumonias, Mycoplasma):ab,ti,kw OR (Refractory Mycoplasma ovipneumoniae Infection):ab,ti,kw OR (Refractory Mycoplasma ovipneumoniae Infections):ab,ti,kw OR (Refractory Mycoplasma pneumoniae Infection):ab,ti,kw OR (Refractory Mycoplasma pneumoniae Infections):ab,ti,kw OR (Refractory Mycoplasma dispar Infection):ab,ti,kw OR (Refractory Mycoplasma dispar Infections):ab,ti,kw

#3: #1 or #2

#4:L-Lactate Dehydrogenase

#5:(LDH):ab,ti,kw OR (Dehydrogenase, L-Lactate):ab,ti,kw OR (L Lactate Dehydrogenase):ab,ti,kw OR (Lactate Dehydrogenase):ab,ti,kw OR (Dehydrogenase, Lactate):ab,ti,kw

#6: #4 or #5

#7:(sensitiv):ab,ti,kw OR (sensitivity and specificity):ab,ti,kw OR (predictive):ab,ti,kw OR (predictive value of tests):ab,ti,kw OR (accuracy):ab,ti,kw

#8:#3 and #6 and #7

Web of science

#1 TS=(Pneumonia, Mycoplasma or Refractory Mycoplasma pneumoniae pneumonia or Refractory Pneumonia, Primary Atypical or Refractory Atypical Pneumonia, Primary or Refractory Atypical Pneumonias, Primary or Refractory Pneumonias, Primary Atypical or Refractory Primary Atypical Pneumonia or Refractory Primary Atypical Pneumonias or Refractory Mycoplasma Pneumonia or Refractory Mycoplasma Pneumonias or Refractory Pneumonias, Mycoplasma or Refractory Mycoplasma ovipneumoniae Infection or Refractory Mycoplasma ovipneumoniae Infections or Refractory Mycoplasma pneumoniae Infection or Refractory Mycoplasma pneumoniae Infections or Refractory Mycoplasma dispar Infection or Refractory Mycoplasma dispar Infections)

#2 TS=(L-Lactate Dehydrogenase or LDH or Dehydrogenase, L-Lactate or L Lactate Dehydrogenase or Lactate Dehydrogenase or Dehydrogenase, Lactate)

#3 TS=(sensitiv or sensitivity and specificity or predictive or predictive value of tests or accuracy)

#4 #1 AND #2 AND #3

CBM:

#1 "肺炎, 支原体"[不加权:扩展]

#2 难治性支原体肺炎
#3 难治性肺炎支原体肺炎

#4 (难治性肺炎支原体肺炎) OR (难治性支原体肺炎) OR ("肺炎, 支原体"[不加权:扩展])

#5 "L-乳酸脱氢酶"[不加权:扩展]

#6 乳酸脱氢酶

#7 LDH

#8 (LDH) OR (乳酸脱氢酶) OR ("L-乳酸脱氢酶"[不加权:扩展])

#9 "诊断"[不加权:扩展]

#10 ("诊断"[不加权:扩展]) AND ((LDH) OR (乳酸脱氢酶) OR ("L-乳酸脱氢酶"[不加权:扩展])) AND ((难治性肺炎支原体肺炎) OR (难治性支原体肺炎) OR ("肺炎, 支原体"[不加权:扩展]))

CNKI:

主题：（肺炎, 支原体 or 难治性肺炎支原体肺炎 or难治性支原体肺炎）and 摘要：（乳酸脱氢酶 or LDH）and 摘要：（诊断）

万方:

主题：（肺炎, 支原体 or 难治性肺炎支原体肺炎 or难治性支原体肺炎）and 摘要：（乳酸脱氢酶 or LDH）and 摘要：（诊断）

VIP:

（肺炎, 支原体 or 难治性肺炎支原体肺炎 or难治性支原体肺炎）and 摘要：（乳酸脱氢酶 or LDH）and 摘要：（诊断）
